# ZNF692 regulates nucleolar morphology by interacting with NPM1 and modifying its self-assembly properties

**DOI:** 10.1016/j.jbc.2024.105773

**Published:** 2024-02-19

**Authors:** Isabella N. Brown, Arlene Levario, Chunhui Jiang, Weronika Stachera, Enrique Rodriguez, Yi-Heng Hao, Jeffrey B. Woodruff, M.Carmen Lafita-Navarro, Maralice Conacci-Sorrell

**Affiliations:** 1 Department of Cell Biology, University of Texas Southwestern Medical Center, Dallas, Texas, USA; 2 Hamon Center for Regenerative Science and Medicine, University of Texas Southwestern Medical Center, Dallas, Texas, USA; 3 Harold C. Simmons Comprehensive Cancer Center, University of Texas Southwestern Medical Center, Dallas, Texas, USA

**Keywords:** nucleolus, protein assembly, condensates, NPM1, ZNF692

## Abstract

The nucleolus, a membrane-less organelle, is responsible for ribosomal RNA transcription, ribosomal RNA processing, and ribosome assembly. Nucleolar size and number are indicative of a cell's protein synthesis rate and proliferative capacity, and abnormalities in the nucleolus have been linked to neurodegenerative diseases and cancer. In this study, we demonstrated that the nucleolar protein ZNF692 directly interacts with nucleophosmin 1 (NPM1). Knocking down *ZNF692* resulted in the nucleolar redistribution of NPM1 in ring-like structures and reduced protein synthesis. Purified NPM1 forms spherical condensates *in vitro* but mixing it with ZNF692 produces irregular condensates more closely resembling living cell nucleoli. Our findings indicate that ZNF692, by interacting with NPM1, plays a critical role in regulating nucleolar architecture and function in living cells.

The nucleolus is a membrane-less organelle playing a critical role in almost every step of ribosome biogenesis. Efficient ribosome biogenesis is critical for translation and cell growth (1). Thus, misregulation of nucleolar activity can cause devastating human diseases from ribosomopathies to cancer (1, 2, 3, 4, 5, 6, 7, 8, 9). The nucleolus contains 3 functionally and spatially distinct compartments: the fibrillar center, the dense fibrillar component, and the granular component. In the fibrillar center, pre-ribosomal RNA (rRNA) is synthesized as a single transcript that undergoes several maturation steps. Simultaneously, the pre-rRNA is combined with ribosomal proteins forming 40S small ribosomal subunits with 18S rRNA and 60S large ribosomal subunits with 5.8S, 28S rRNAs, and 5S rRNA (transcribed separately) (10, 11). Mature 60S and 40S subunits form the 80S ribosome in the cytoplasm.

The 3 nucleolar compartments are formed by liquid-liquid phase separation where the concentration of specific multivalent interactions create liquid-like unmixable phases. *In vitro* mixing of fibrillarin (FBL), a component of the dense fibrillar, and nucleophosmin 1 (NPM1), a key component of the granular component, is sufficient to create a multilayered droplet that recapitulates the nucleolar sub-compartments (12). Additionally, heterotypic interactions between main nucleolar scaffold proteins and rRNA modulate the assembly of nucleolar-like structures (12, 13, 14). Although spherical nucleoli, which resemble *in vitro* assembled nucleoli, have been observed in living cells including in *C. elegans* and *Xenopus laevis* embryos (15, 16), most functional nucleoli in mammalian cells do not appear spherical, but rather as grainy structures with irregular boundaries. This disconnection between simplified *in vitro* systems and more complex *in vivo* systems raises the possibility that additional unidentified proteins act to modulate the final structure of the nucleolus.

Our group recently discovered a novel nucleolar component, the Zn finger protein ZNF692 as a MYC-induced nucleolar scaffold (17). ZNF692 localizes in the granular component where it forms a hub that facilitates the final steps of 40S subunit biogenesis (17). Here, we demonstrate that ZNF692 directly interacts with NPM1 and influences nucleolar architecture. In the presence of ZNF692, nucleoli exhibit an irregular morphology, with NPM1 evenly distributed, a characteristic of highly active nucleoli. *In vitro* experiments reveal that ZNF692 alters the phase-separated droplets of NPM1.

## Results and discussion

### ZNF692 directly interacts with NPM1 in the nucleolus

**Figure 1:**
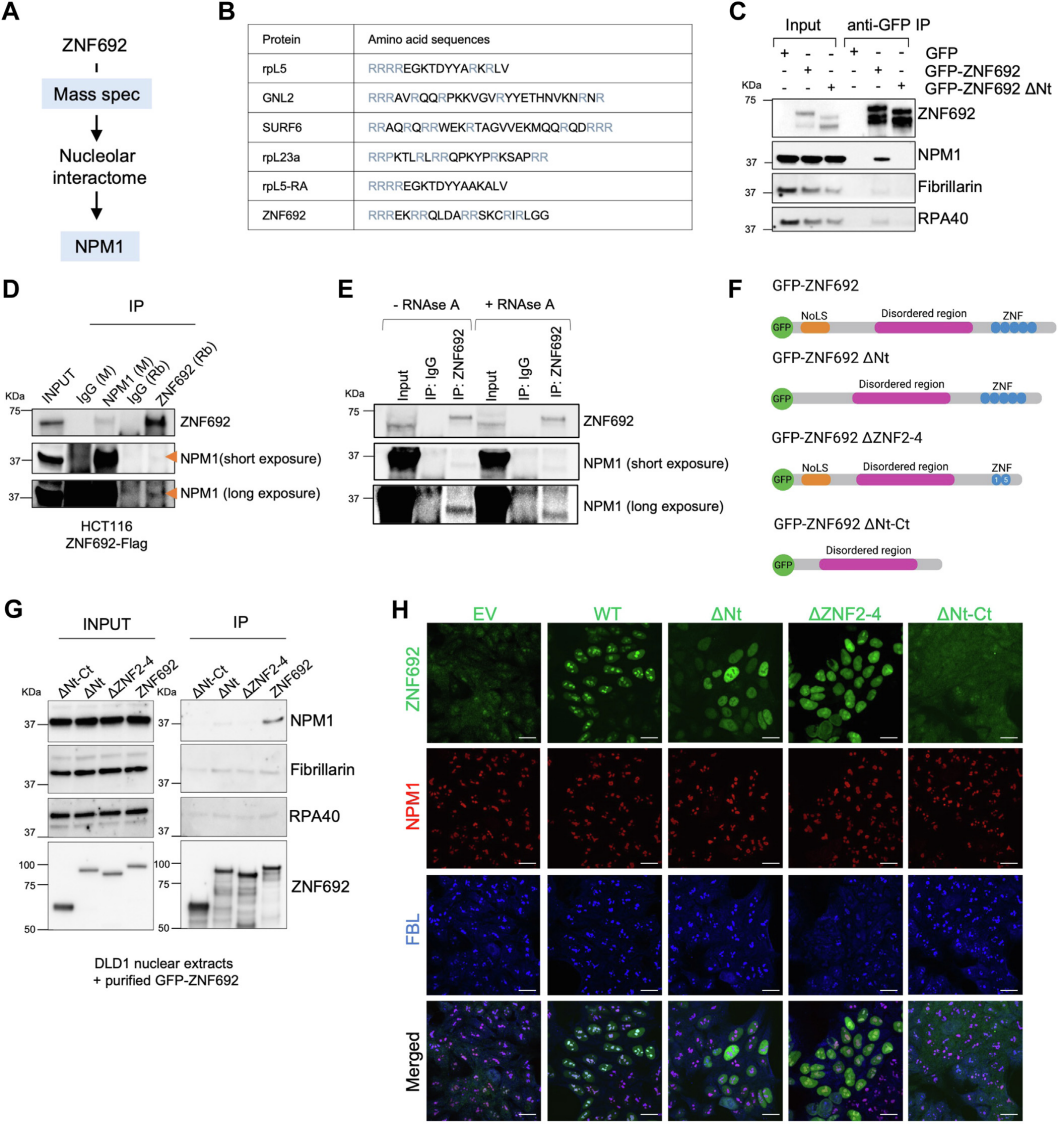
**ZNF692 directly interacts with NPM1 in the nucleolus.***A*, schematic of ZNF692 pulldown proteomics. *B*, R-motif sequences of NPM1 interactors compared to the ZNF692 Nt domain (NoLS). *C*, immunoprecipitation of HCT116 cells transfected with GFP, GFP-ZNF692 or GFP-ZNF692 ΔNt and immunoblotted for ZNF692, NPM1, FBL, and RPA40. *D*, ZNF692 and NPM1 immunoprecipitation in HCT116 ZNF692-Flag. *E*, ZNF692 and NPM1 immunoprecipitation in HCT116 ZNF692-Flag (with and without RNase A). *F*, schematic of recombinant GFP-ZNF692. GFP-ZNF692 (ZNF692 amino acids 1–519), GFP-ZNF692 ΔNt (lacking ZNF692 amino acids 3–33), GFP-ZNF692 ΔZNF2-4 lacking zinc fingers 2 to 4 (lacking ZNF692 amino acids 360–439), and GFP-ZNF692 ΔNt-Ct (lacking amino acids 1–97 and 320–519). *G*, immunoprecipitation of the recombinant ZNF692 in (*F*) with DLD1 nuclear extracts followed by immunoblot for NPM1, FBL, and RPA40. *H*, Immunofluorescence of ZNF692, NPM1, and FBL in DLD1 cells expressing ZNF692 WT or mutants. Scale bar = 20 μm.

Using mass spectrometry (17), we found that ZNF692 interacted with NPM1 (Fig. 1*A*) (17). *In silico* analyses of ZNF692 identified an R-rich linear motif within its N-terminus (Nt) (Fig. 1*B*). R-rich motifs mediate the interaction of rpL5, SURF6, GNL2, and rpL23a with NPM1 (Fig. 1*B*) (13). Immunoprecipitations (IP) of ZNF692 from lysates of HCT116 cells expressing GFP-tagged ZNF692, ZNF692 lacking the N-terminal domain (ΔNt), or GFP confirmed the interaction between ZNF692 WT and NPM1 (Fig. 1*C*), consistent with the observed colocalization of ZNF692 with NPM1 in the granular component of the nucleolus (17). ZNF692 interaction with NPM1 was reduced by the deletion of ZNF692’s Nt domain (Fig. 1*C*), which contains the nucleolar localization signal (NoLS) and the R-rich motif. Notably, ZNF692 exhibited no strong interaction with FBL (a dense fibrillar component marker) or with RPA40 (a fibrillar center marker). We further established the interaction between ZNF692 and NPM1 by reciprocal IP of NPM1 and ZNF692 in cells stably expressing *ZNF692-*Flag (Fig. 1*D*). Although our previous study demonstrated that the interaction of ZNF692 with rRNA processing factors requires RNA (17), we found that the interaction of ZNF692 with NPM1 was unaffected by adding RNAse A to pre-IP lysates (Fig. 1*E*).

To define the ZNF692 domains interacting with NPM1, we used recombinant GFP-ZNF692, GFP-ZNF692 ΔNt (lacking the NoLS), GFP-ZNF692 ΔZNF2-4 (lacking Zn fingers 2, 3 and 4), and GFP-ZNF692 ΔNt-Ct (lacking the NoLS and C-terminal region) (Fig. 1*F*) (17). *In vitro* IP of GFP-tagged proteins mixed with DLD1 nuclear extracts demonstrated that GFP-ZNF692, but none of the mutants, had the ability to interact with NPM1, indicating that the Nt or C-terminal (Ct) domains are both required for the interaction with NPM1 (Fig. 1*G*). FBL and RPA40 did not co-IP with purified ZNF692 *in vitro*, demonstrating the specificity of ZNF692 and NPM1 interaction and confirming the *in vivo* IPs (Fig. 1*C*). In agreement with the interaction experiments, ZNF692 and ZNF692 ΔZNF2-4 (to a lesser extent), co-localized with NPM1 in the granular component of the nucleolus. ZNF692 ΔNt localized in the nucleoplasm while ZNF692 ΔNt-Ct was present in the nucleoplasm and cytoplasm (Fig. 1*H*). These results indicate that the presence of both Ct and Nt regions optimally targets ZNF692 to the nucleolus and thus puts ZNF692 into proximity with NPM1.

### ZNF692 depends on NPM1 to promote protein synthesis

**Figure 2:**
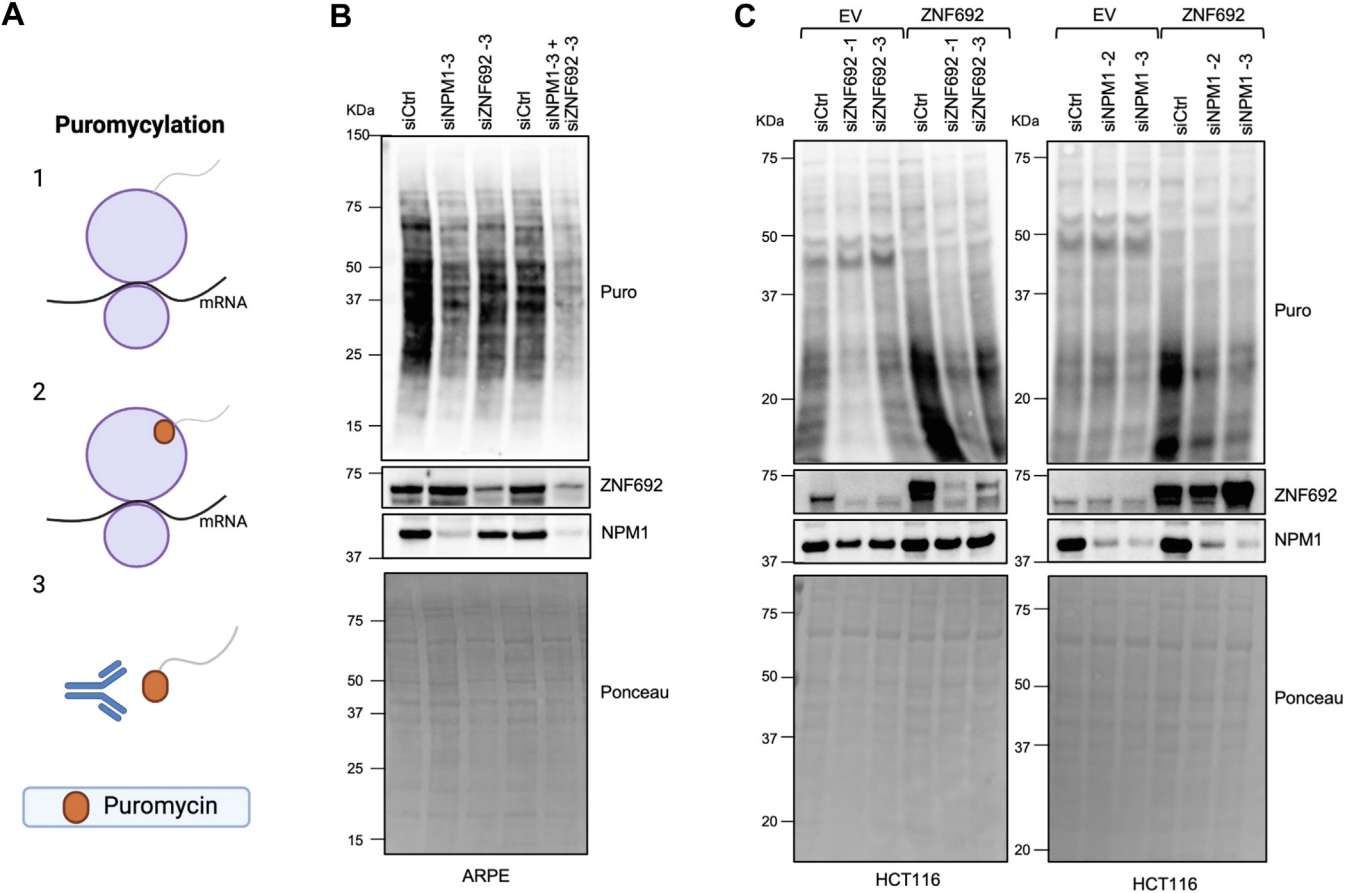
**ZNF692 depends on NPM1 to promote protein synthesis.***A*, puromycylation: Puromycing incorportates into translating peptides. Puromycin-labeled peptides can be detected by Western blot using an anti-puromycin antibody. *B*, puromycylation in ARPE cells 72 h after transfection with control, NPM1 or ZNF692 siRNA. Immunoblot for indicated antibodies. *C*, puromycylation in HCT116 cells expressing *ZNF692* or empty vector (EV) after 72 h of control, ZNF692 and NPM1 siRNA transfection. Immunoblot for indicated antibodies.

To assess if the interaction between ZNF692 and NPM1 is necessary for ZNF692-dependent translation, we knocked down (KD) *NPM1* and *ZNF692* in retinal epithelial cells (ARPE) and in cells stably expressing either an empty vector or *ZNF692* and measured translation by puromycylation (Fig. 2*A*). Individual *NPM1* and *ZNF692* KD in ARPE cells decreased protein synthesis. However, the combined *NPM1* and *ZNF692* KD had an even more pronounced effect (Fig. 2*B*). Ectopic expression of *ZNF692* increased protein synthesis in HCT116 and DLD1 cells (Figs. 2*C*, and S1*A*), while *ZNF692* KD decreased it (Fig. 2*C*). Interestingly, *NPM1* KD reduced protein synthesis in HCT116 cells stably expressing *ZNF692* but did not reduce protein synthesis in empty vector cells (Fig. 2*C*, right panel). The fact that *NPM1* KD only diminished protein translation in cells overexpressing *ZNF692* implies that the enhanced protein translation associated with ZNF692 relies, to some extent, on NPM1. Unexpectedly, *NPM1* overexpression did not increase translation (Fig. S1*B*). Nevertheless, *NPM1* KD reduced proliferation of DLD1 and HCT116 cells independently of ZNF692 levels (Fig. S1, *C* and *D*). Overexpression of *ZNF692* did not compensate for *NPM1* KD in cell proliferation (Fig. S1, *C* and *D*). Altogether, these results indicate that NPM1 is required for the increased protein synthesis driven by ZNF692.

### ZNF692 alters the morphology of NPM1 droplets

**Figure 3:**
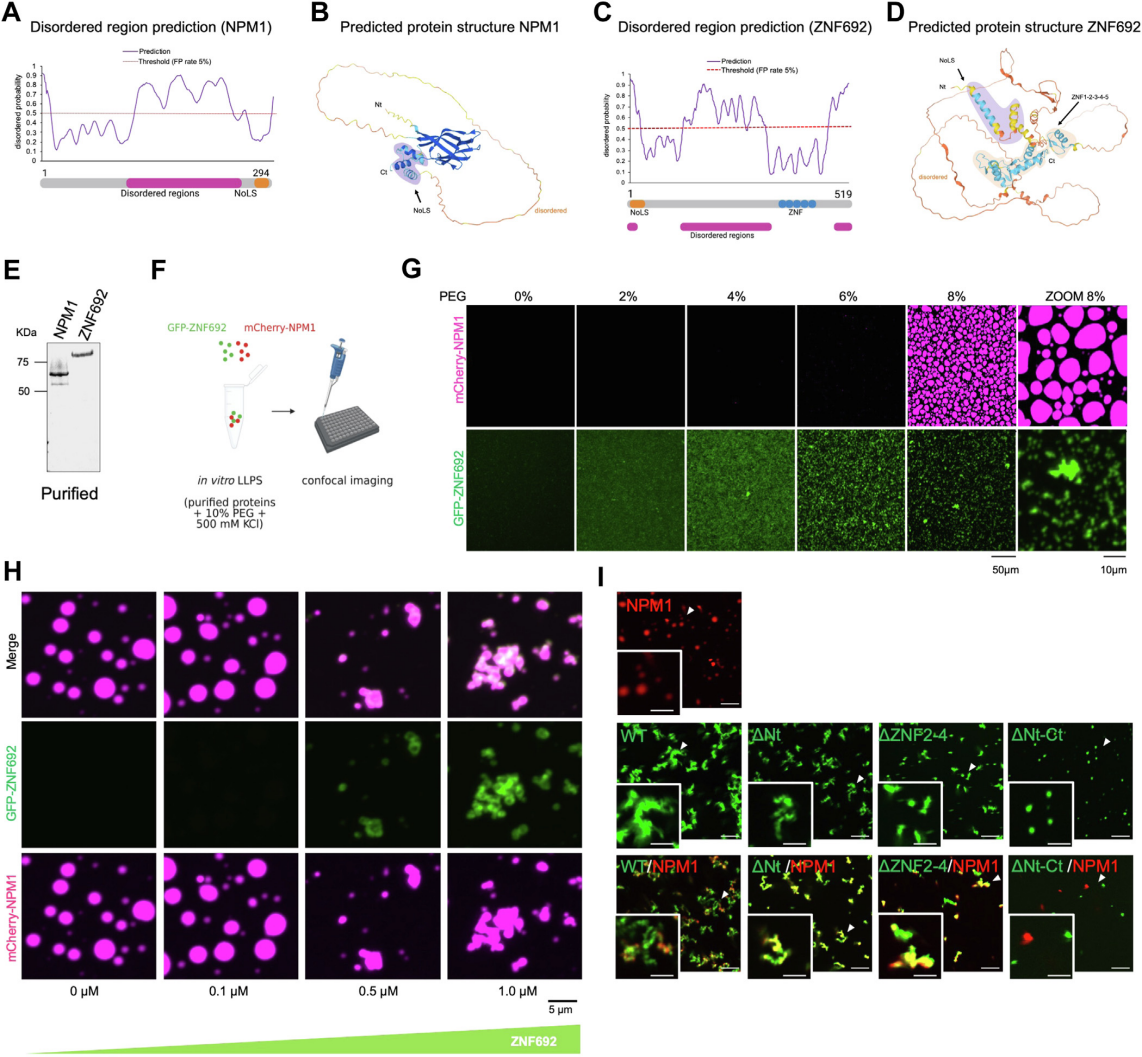
**ZNF692 alters the morphology of NPM1 droplets.***A*, NPM1 predicted disordered region identified with Protein DisOrder prediction System (PrDOS). *B*, NPM1 predicted protein structure using AlphaFold. *C*, ZNF692 predicted disordered regions identified with Protein DisOrder prediction System (PrDOS). *D*, ZNF692 predicted protein structure using AlphaFold. *E*, coomassie-stained gel of purified mCherry-NPM1 and GFP-ZNF692. *F*, schematic of the *in vitro* complex formation experiments using GFP-ZNF692 and mCherry-NPM1 alone or in combination. *G*, self-assembly of 5 μM GFP-ZNF692 or mCherry-NPM1 in the presence of increasing PEG amounts. *H*, self-assembly of 5 μM purified mCherry-NPM1 in 10% PEG and increasing GFP-ZNF692 amounts. *I*, self-assembly of 5 μM purified GFP-ZNF692 WT or mutants and mCherry-NPM1 in 10% PEG. Scale bar = 10 μm. Scale bar insert picture = 5 μm.

Protein DisOrder Prediction System (18) and AlphaFold (19) showed that NPM1 and ZNF692 have a central disordered region (Fig. 3, *A*–*D*). ZNF692 also has two short, disordered regions in the Nt and Ct domains (Fig. 3*C*). AlphaFold-predicted NPM1 and ZNF692 structures (Fig. 3, *B* and *D*) suggested that the central disordered region facilitate the proximity between the Nt and Ct domains (Fig. 3, *B* and *D*). NPM1 has been shown to self-assemble into spherical structures *in vitro* (13), given that ZNF692 and NPM1 interacted (Fig. 1), we asked whether ZNF692 and NPM1 affected each other’s self-assembly properties.

NPM1 and ZNF692 (Fig. 3*E*) were incubated in KCl containing buffer with increasing concentrations of the organic polymer polyethylene glycol (PEG) to simulate the crowded conditions found inside cells (Fig. 3, *F* and *G*). NPM1 exhibited no ability to undergo liquid-liquid phase separation independently but only in the presence of 8% or higher PEG. On the other hand, even with elevated PEG levels, ZNF692 did not undergo phase separation into spherical droplets. Instead, when incubated with 4% or more PEG, ZNF692 formed irregular aggregates (Fig. 3*G*). Interestingly, adding ≥0.5 μM ZNF692 modified NPM1 droplets towards irregular aggregates (Fig. 3*H*) that better resembled the morphology of active nucleoli of living cells (Fig. 1*H*). GFP-EB1, which is known to self-assembly (20), did not disrupted NPM1 droplets (Fig. S2*A*). Interestingly, the R-rich peptides of the previously reported NPM1 interactors rpL5, SURF6, GNL2, and rpL23a, do not alter the spherical morphology of NPM1 droplets (13), thus indicating that the ability of ZNF692 to modify liquid-like NPM1 complexes is specific.

Removing the Nt or Zn fingers 2 to 4 reduced the ability of ZNF692 to alter NPM1’s assembly (Fig. 3*I*). In contrast, removing both the Nt and the Ct domains of ZNF692 allowed for its self-assembly into spherical droplets that did not mix with NPM1 (Fig. 3*I*). These results indicate that the central domain of ZNF692 is capable of self-assembly, a property likely modified by the Nt and Ct domains. To better mimic cellular crowding conditions, we mixed GFP-ZNF692 and mCherry-NPM1 with HCT116 nuclear extracts (Fig. S2*B*). Surprisingly, in these conditions neither ZNF692 nor NPM1 formed round condensates alone or when incubated together (Fig. S2*B*), but they oligomerized into irregular structures, suggesting that additional factors present in nuclear extracts modify the phase separation properties of NPM1 and ZNF692.

### NPM1 N-terminal domain is necessary to colocalize and interact with ZNF692 in the nucleolus

**Figure 4:**
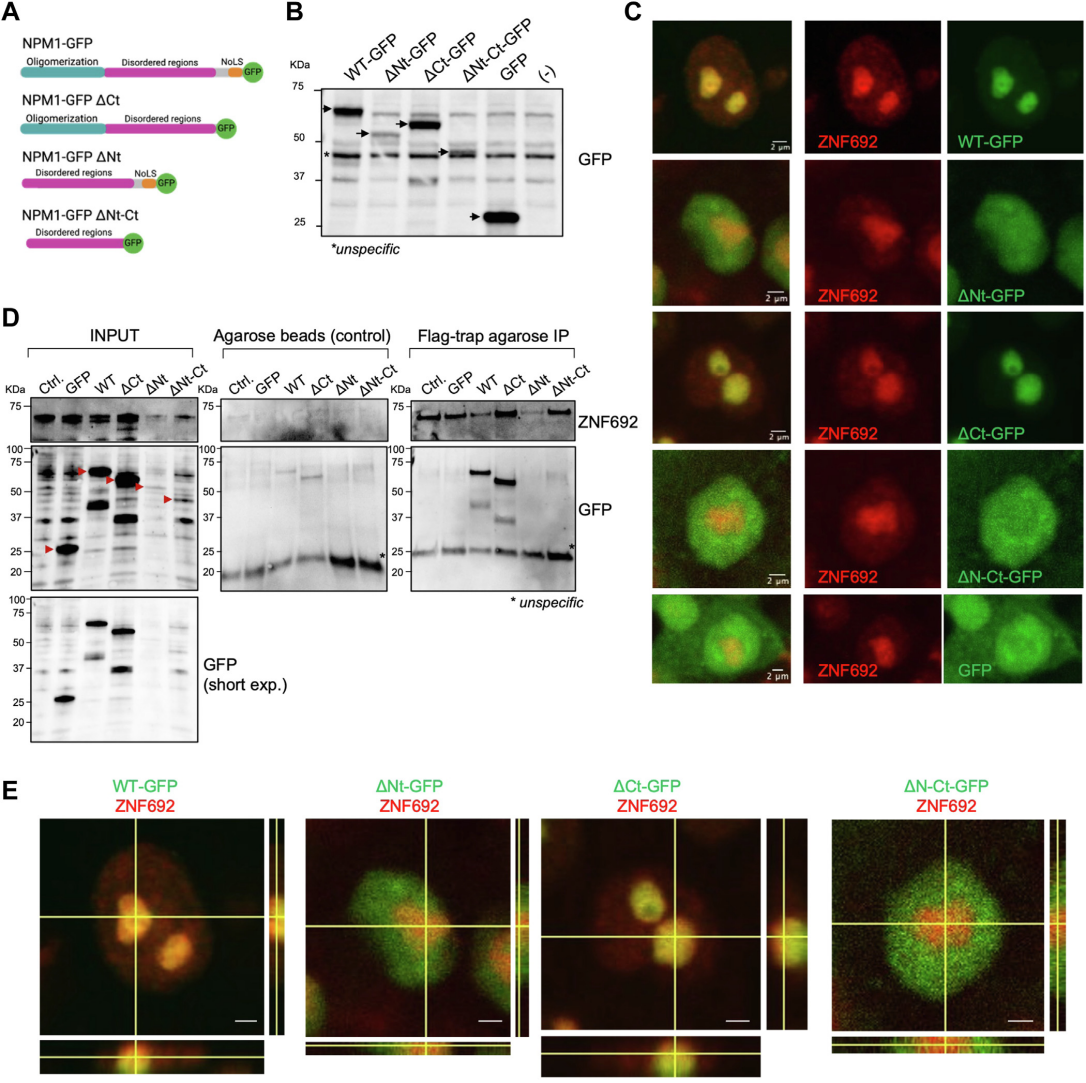
**NPM1 N-terminal domain is necessary to colocalize and interact with ZNF692 in the nucleolus.***A*, NPM1-GFP: wild type NPM1 (amino acids 1–305), and NPM1 mutants: NPM1-GFP ΔCt (lacking amino acids 245–305), NPM1-GFP ΔNt (lacking amino acids 1–117), and NPM1-GFP ΔNt-ΔCt (lacking amino acids 1–117 and 245–305). *B*, WB showing NPM1-GFP expression in HCT116 ZNF692-Flag. *C*, immunofluorescence of NPM1-GFP mutants and ZNF692 in HCT116 ZNF692-Flag cells. Max intensity from z-stack images is shown. *D*, ZNF692-Flag immunoprecipitation with Flag-trap beads or agarose beads (control) in HCT116 ZNF692-Flag cells stably expressing NPM1-GFP or GFP constructs or control (no construct). *E*, orthogonal views from z-stack images in (*C*) showing partial colocalization of WT and ΔCt NPM1 and ZNF692 (*yellow area*). Scale bar = 2 μm.

To define the domains on NPM1 that enable its interaction with ZNF692, we generated vectors to express GFP-tagged NPM1 (NPM1-GFP) and mutants lacking 245 to 305 amino acids including the NoLS region (NPM1-GFP ΔCt), lacking oligomerization region (NPM1-GFP ΔNt), and lacking oligomerization and NoLS regions (NPM1-GFP ΔNt-ΔCt) (Fig. 4, *A* and *B*) in HCT116 that also expressed *ZNF692*-Flag. NPM1-GFP colocalized with ZNF692 in the nucleolus, while NPM1-GFP ΔNt and NPM1-GFP ΔNt-ΔCt localized to the nucleoplasm and separated from ZNF692 (Figs. 4*C* and S3*A*). NPM1-GFP ΔCt exhibited an unexpected nucleolar localization, even in the absence of a NoLS. This phenomenon may be attributed to potential interactions with specific binding partners that facilitate its targeting to the nucleolus. Consistent with their nucleolar co-localization, NPM1-GFP and NPM1-GFP ΔCt, but not the ΔNt and ΔNt-ΔCt, were co-IPed with ZNF692 (Figs. 4*D* and S3*B*). The ΔNt and ΔNt-ΔCt NPM1 mutants were expressed at lower levels. Orthogonal views obtained through z-stack immunofluorescence (IF) of ZNF692 and NPM1 confirm their partial colocalization in the nucleolus (Fig. 4, *C* and *E*).

### NPM1 distribution within the nucleolus is affected by the presence of ZNF692

**Figure 5:**
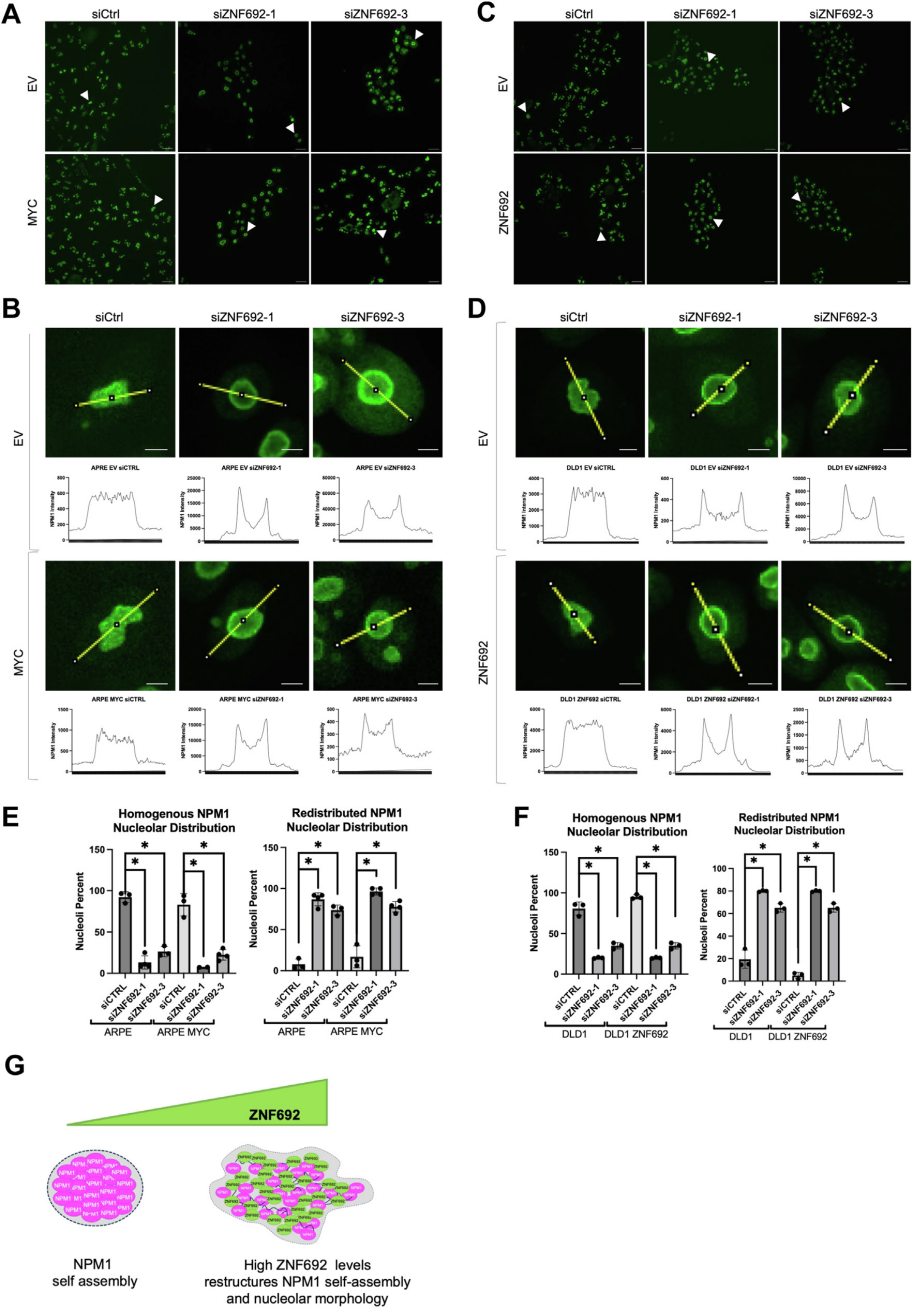
**NPM1 distribution within the nucleolus is affected by the presence of ZNF692.***A*, immunofluorescence of NPM1 in ARPE and ARPE-MYC 72 h after transfection with control or ZNF692 siRNAs. Scale bar = 20 μm. *B*, example of intensity profile of nucleolar NPM1 indicated by white arrow in Figure 5*A*. Scale bar = 4 μm. *C*, immunofluorescence of NPM1 in DLD1 and DLD1 ZNF692 cells transfected with control or ZNF692 siRNAs. Scale bar = 20 μm. *D*, example of the intensity profile of nucleolar NPM1 indicated by white arrow in Figure 5*C*. Scale bar = 4 μm. *E*, percentage of cells with homogenous or redistributed NPM1 from (*A*). Each data point represents NPM1 distribution percentage within an image. N = 100 cells from three experiments. *F*, percentage of cells with homogenous or redistributed NPM1 from (*C*). Each data point represents NPM1 distribution percentage within an image. N = 100 cells from 3 experiments. *G*, working model. NPM1 self-assemblies into spherical structures. In the presence of ZNF692, ZNF692 interacts with NPM1 restructuring NPM1 self-assembly and allowing for a more interconnected and active nucleoli.

Given that NPM1 is the major regulator of nucleolar assembly and that ZNF692 alters the assembly properties of NPM1 *in vitro*, we asked whether ZNF692 regulates the nucleolar distribution of NPM1. First, we confirmed that *ZNF692* KD does not affect FBL distribution (Fig. S4), in accordance with the lack of interaction between FBL and ZNF692. To determine the importance of ZNF692 for the nucleolar distribution of NPM1, we used ARPE cells expressing empty vector or ARPE-MYC, which express high levels of ZNF692 (Fig. S5*A*). *ZNF692* KD in ARPE and ARPE-MYC cells (Fig. S5, *A* and *B*) altered NPM1 distribution in the nucleolus, leading to the formation of halo-like structures as visualized by IF (Figs. 5, *A*, *B* and *E*, S5, *C* and *D*, S7 and S8). Furthermore, we examined the effects of *ZNF692* KD on NPM1 distribution in DLD1 cells either expressing empty vector or ectopically expressing *ZNF692*. Our experiments revealed that while *ZNF692* KD in these cells did not change nucleolar number (Fig. S6*A*), both cell lines had a significant change in NPM1 distribution upon acute *ZNF692* silencing as shown by IF (Fig. 5, *C*, *D* and *F*, S6*B*, S9, S10) indicating that ZNF692 is necessary for the homogenous NPM1 distribution within the nucleolus. Quantification of Figure 5, *A* and *B* demonstrated a significant redistribution of NPM1 (Fig. 5, *E* and *F*, representative images in Figs. S7–S10). Given that ZNF692 depletion led to NPM1 redistribution towards the nucleolar periphery, a hallmark of nucleolar stress (6), we propose a model in which ZNF692 by interacting with NPM1 maintains active nucleolar structure and function (Fig. 5*G*). It is possible that ZNF692 acts as an emulsifier, effectively preventing the liquid phase separation of NPM1.

## Experimental procedures

### Cell culture

Human colorectal carcinoma cells HCT-116 (ATCC- CCL-247) and DLD-1 (ATCC- CCL-221) were purchased from ATCC. Human Retinal pigment epithelial cell line ARPE-19 was obtained from the Schmid lab, UTSW. All cells were cultured in Dulbecco's Modified Eagle Medium (DMEM) supplemented with 10% Fetal Bovine Serum (FBS), 100 U/ml Penicillin-Streptomycin. Cell viability was measured with crystal violet staining following a previously published method (17). For puromycylation analysis, after overnight serum starvation, cells were placed in serum-containing media for 6 h and puromycin was added at 20 μg/ml for DLD1 and 10 μg/ml for HCT116 cells for 2 h. Cells were lysed with RIPA buffer for WB using anti-puromycin antibody.

### Cell transfection and infection

1 × 10^5^ cells were seeded and transfected with siRNA (see Table S1) and lipofectamine RNAiMAX (Invitrogen) and analyzed 72 h after transfection. Ectopic expression of ZNF692 wild-type (WT) and mutants and NPM1-GFP WT and mutants was achieved through retroviral or lentiviral infection respectively. Retro- and lentiviral particles were produced in 293T cells with plasmids containing the construct of interest together with Anpho (retrovirus) or psPAX2 and pMDG2 (lentivirus) virus packaging plasmids. For viral infection, 1 × 10^5^ cells were seeded with polybrene (10 μg/ml), viral supernatant, and cell culture media. Post-infection, cells were selected with hygromycin (1 mg/ml) or gentamicin (1 mg/ml).

### Plasmids

pBabe-hygro-ZNF692 WT or mutants were constructed by cloning ZNF692 sequences from pCMV6-Entry-ZNF692 (Origene RC200163) into pBabe-hygro vectors. For *in vitro* purified protein, coding sequences of ZNF692 and NPM1 were cloned into pOCC29-TEV-GFP and pOCC206-mCherry (17). WT NPM1-GFP (Uniprot: A0A7I2V579), ΔNt NPM1-GFP (lacking 1–117 amino acids), ΔCt-NPM1 (lacking 245–305 amino acids), ΔNt-Ct NPM1 (lacking 1–117 and 245–305 amino acids), and GFP constructs on pLV-CMV were designed and purchased from VectorBuilder.

### Immunofluorescence

Cells were fixed in 4% paraformaldehyde for 20 min, permeabilized with 0.1% Triton X-100 for 20 min, then blocked for 1 h in 1% BSA-PBS. Primary antibodies were incubated overnight at 4 °C, then washed with PBS and incubated for 1 h with secondary antibodies at room temperature followed by 3 PBS washes including one wash with 4′,6-diamidino-2-phenylindole (DAPI). Coverslips were mounted and images were taken with a Zeiss LSM780 fluorescent or Spinning disk Nikon microscope. ImageJ was used for analysis. The nucleolar NPM1 intensity profile was graphed to assess the homogenous or redistributed pattern within an image (N = 100 cells counted among 3 biological replicates). Each data point reflects the percentage of one image per experiment. The percentage of cells with <3 nucleoli or ≥3 nucleoli was calculated per image and graphed as a data point.

### Western blot

Cells were lysed in RIPA buffer (50 mM Tris-HCl (pH 8.0), 5 mM EDTA, 150 mM NaCl, 0.5% Sodium deoxycholate, 0.1% Nonidet P-40, 0.1% SDS) or 10 mM Tris-HCl pH 8.0, 50 mM NaCl, 0.1% NP-40 (Fig. 4*B*) and proteinase inhibitors. Proteins were separated by SDS–polyacrylamide gel, transferred to nitrocellulose membrane, and probed with antibodies (Table S1).

### Condensate formation

Recombinant ZNF692 and NPM1 were generated as previsouly described (17). Purified proteins were diluted to 5 μM in 500 mM KCl and polyethylene glycol (PEG, MW 3350; SIGMA), incubated 5 min at room temperature and transferred to a glass-bottom 96 well plate. Alternatively, 5 μM final concentration of GFP-ZNF692 or mCherry-NPM1 were mixed with HCT116 nuclear extracts. Cells were lysed in 10 mM Hepes, 20 mM KCl and 0.1% NP-40 and nuclear pellet was lysed with PBS and sonication in the presence of RNAse and protease inhibitors. Images were acquired by confocal microscopy.

### Immunoprecipitation

Cells were harvested in lysis buffer (10 mM Tris-HCl pH 8.0, 50 mM NaCl, 0.5% NP-40, protease inhibitors), sonicated and centrifuged at 12,000*g* at 4 °C for 20 min and supernatants were used. 10% of each sample was used as input, the remainder was used for IP. For Figure 1*A*, HCT116 cells expressing ZNF692-Flag were transiently transfected with GFP, GFP-ZNF692 or GFP-ZNF692 ΔNt and lysates were incubated with GFP-trap beads (Proteintech, gtma). For Figure 1, *D* and *E* HCT116 ZNF692-Flag lysates were immunoprecipitated with Protein G magnetic beads and primary antibodies (Table S1) overnight. For Figure 4*D* and Fig. S4, ZNF692-Flag from HCT116 ZNF692-Flag cells stably expressing GFP, NPM1-GFP, or mutants NPM1-GFP (ΔNt, ΔCt, ΔNt-Ct) lysates were immunoprecipitated with DYKDDDDK Fab-trap beads (Proteintech, ffa) and protein A/G PLUS-Agarose (Santacruz, sc-2003) was used as negative control. For Figure 1*G*, nuclear extracts were isolated from DLD1 cells as described (17). Purified GFP-ZNF692 WT or mutant recombinant proteins were incubated with GFP-trap beads and incubated with the nuclear extracts overnight. All immunoprecipitates were washed with lysis buffer 3 to 5 times for 10 min and subjected to Western blot.

### Statistical analysis

All statistical analyses were performed using a two-tailed student *t* test. *P* ≤ 0.05 was considered statistically significant. All values are reported as mean ± SD.

## Data availability

All data used for this study is available within the manuscript.

## Supporting information

This article contains supporting information.

## Conflict of interest

The authors declare that they have no conflicts of interest with the contents of this article.

## Supporting information

Supporting Figure S1 **NPM1 and ZNF692 cooperate to promote protein synthesis.***A*, Puromycylation of DLD1 empty vector or ZNF692-WT. 3 independent experiments are shown. *B*, Puromycylation of NPM1-GFP WT or GFP expressing HCT116 ZNF692-Flag cells. *C*, Relative proliferation of empty vector (EV) and ZNF692 expressing DLD1 cells after 72 h transfection with control or NPM1 siRNAs. Western blot for NPM1, ZNF692, and actin. *D*, Relative proliferation of HCT116 EV (empty vector) and HCT116 ZNF692 cells after 72 h transfection with control or NPM1 siRNAs. Western blot for NPM1, ZNF692, and tubulin. **Supporting Figure S2ZNF692 alters the morphology of NPM1 droplets.***A*, self-assembly of 5 μM GFP-EB1 and mCherry-NPM1 in 8% PEG 500 mM KCl. Scale bar = 10 μm. *B*, self-assembly of 5 μM mCherry-NPM1 and GFP-ZNF692 in HCT116 nuclear extracts. Scale bar = 10 μm. **Supporting Figure S3NPM1 N-terminal domain is necessary to colocalize and interact with ZNF692 in the nucleolus.***A*, ZNF692 immunofluorescence in HCT116 *ZNF692*-Flag cells expressing *NPM1*-GFP and mutants. *B*, ZNF692-Flag immunoprecipitation with Flag-trap beads in HCT116 ZNF692-Flag cells stably expressing NPM1-GFP or GFP constructs or control (no construct). **Supporting Figure S4FBL distribution within the nucleolus is not affected by *ZNF692* KD.** Fibrillarin immunofluorescence in HCT116 cells 3 days after transfection with control or ZNF692 siRNA. Scale bar = 10 μm. **Supporting Figure S5NPM1 distribution within the nucleolus is affected by the presence of ZNF692 in ARPE and ARPE MYC cells.***A*, Western blot of control or *ZNF692* KD in ARPE and ARPE-MYC cells for ZNF692, MYC, and actin. *B*, Western blot of ARPE MYC cells with different ZNF692 siRNA concentrations. *C*, Counted cells used for ARPE and ARPE MYC (Fig. 5). *Blue outlined* images represent images shown in Figure 5*A*. Scale bar = 20 μm. *D*, cells percentage with indicated nucleolar number in APRE EV (empty vector) and ARPE MYC cells (N=>100 cells among 3 to 4 replicates). Data points indicate cell percentage per image. ∗*P* ≤ *0.05*. **Supporting Figure S6NPM1 distribution within the nucleolus is affected by the presence of ZNF692 in DLD1 and DLD1 ZNF692 cells.***A*, cells percentage with indicated nucleolar number in DLD1 EV (empty vector) and DLD1 ZNF692 cells (N=>100 cells among 3 replicates). Data points indicate cell percentage per image. ∗*P* ≤ *0.05*. *B*, counted cells used for DLD1 EV (empty vector) and DLD1 ZNF692 cells (Fig. 5). *Blue outlined* images represent images shown in Figure 5*C*. Scale bar = 20 μm. **Supporting Figure S7NPM1 distribution within the nucleolus in ARPE EV cells.** NPM1 intensity profiles of 4 to 5 extra cells/experiment as in Figure 5*A* in ARPE EV (empty vector) cells. Scale bar = 4 μm. **Supporting Figure S8NPM1 distribution within the nucleolus in ARPE MYC cells NPM1 intensity profiles of 4 to 5 extra cells/experiment as in**Figure 5A**in ARPE MYC cells.** Graphs outlined in blue represent 2 nucleoli/cell (first in *blue* and second in *green*). Scale bar = 4 μm. **Supporting Figure S9NPM1 distribution within the nucleolus in DLD1 EV cells NPM1 intensity profiles of 5 extra cells/experiment as in**Figure 5B**in DLD1 EV (empty vector) cells.** Graphs outlined in *blue* represent 2 nucleoli/cell (first in *blue* and second in *green*). Scale bar = 4 μm. **Supporting Figure S10NPM1 distribution within the nucleolus in DLD1 ZNF692 cells NPM1 intensity profiles of 4 to 5 extra cells/experiment as in**Figure 5B**in DLD1 ZNF692 cells.** Scale bar = 4 μm.

Supporting Table S1 List of Antibodies and siRNA. List of antibodies and siRNA used in this study.
