# Supplementary material for: ZNF692 regulates nucleolar morphology by interacting with NPM1 and modifying its self-assembly properties
Source: J Biol Chem. 2024 Feb 19;300(3):105773. doi: 10.1016/j.jbc.2024.105773 (PMC10956046; doi:10.1016/j.jbc.2024.105773)
Supplement: Supporting Figure S1 — NPM1 and ZNF692 cooperate to promote protein synthesis.A, Puromycylation of DLD1 empty vector or ZNF692-WT. 3 independent experiments are shown. B, Puromycylation of NPM1-GFP WT or GFP expressing HCT116 ZNF692-Flag cells. C, Relative proliferation of empty vector (EV) and ZNF692 expressing DLD1 cells after 72 h transfection with control or NPM1 siRNAs. Western blot for NPM1, ZNF692, and actin. D, Relative proliferation of HCT116 EV (empty vector) and HCT116 ZNF692 cells after 72 h transfection with control or NPM1 siRNAs. Western blot for NPM1, ZNF692, and tubulin. Supporting Figure S2ZNF692 alters the morphology of NPM1 droplets.A, self-assembly of 5 μM GFP-EB1 and mCherry-NPM1 in 8% PEG 500 mM KCl. Scale bar = 10 μm. B, self-assembly of 5 μM mCherry-NPM1 and GFP-ZNF692 in HCT116 nuclear extracts. Scale bar = 10 μm. Supporting Figure S3NPM1 N-terminal domain is necessary to colocalize and interact with ZNF692 in the nucleolus.A, ZNF692 immunofluorescence in HCT116 ZNF692-Flag cells expressing NPM1-GFP and mutants. B, ZNF692-Flag immunoprecipitation with Flag-trap beads in HCT116 ZNF692-Flag cells stably expressing NPM1-GFP or GFP constructs or control (no construct). Supporting Figure S4FBL distribution within the nucleolus is not affected by ZNF692 KD. Fibrillarin immunofluorescence in HCT116 cells 3 days after transfection with control or ZNF692 siRNA. Scale bar = 10 μm. Supporting Figure S5NPM1 distribution within the nucleolus is affected by the presence of ZNF692 in ARPE and ARPE MYC cells.A, Western blot of control or ZNF692 KD in ARPE and ARPE-MYC cells for ZNF692, MYC, and actin. B, Western blot of ARPE MYC cells with different ZNF692 siRNA concentrations. C, Counted cells used for ARPE and ARPE MYC (Fig. 5). Blue outlined images represent images shown in Figure 5A. Scale bar = 20 μm. D, cells percentage with indicated nucleolar number in APRE EV (empty vector) and ARPE MYC cells (N=>100 cells among 3 to 4 replicates). Data points indicate cell percentage p [file mmc1.pdf]

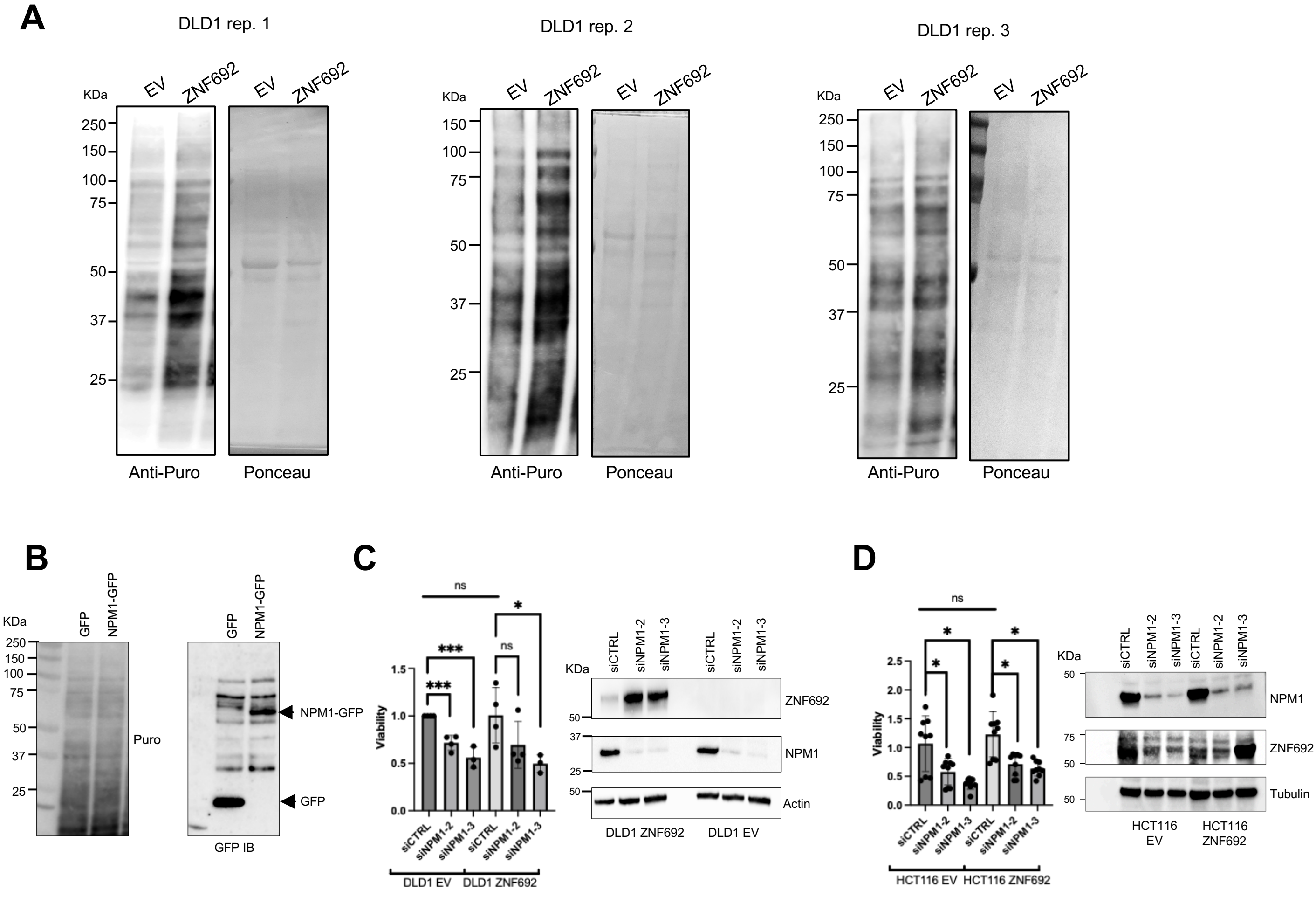
A
DLD1 rep. 1
DLD1 rep. 2
DLD1 rep. 3
KDa
EV
ZNF692
250
150
100
75
50
37
25
Anti-Puro
Ponceau
B
GFP
NPM1-GFP
Puro
NPM1-GFP
GFP
GFP IB
C
ns
***
*
Viability
1.5
1.0
0.5
0.0
siCTRL
siNPM1-2
siNPM1-3
DLD1 EV
DLD1 ZNF692
ZNF692
NPM1
Actin
DLD1 ZNF692
DLD1 EV
D
2.5
2.0
HCT116 EV
HCT116 ZNF692
Tubulin
HCT116 EV
HCT116 ZNF692

Figure S1

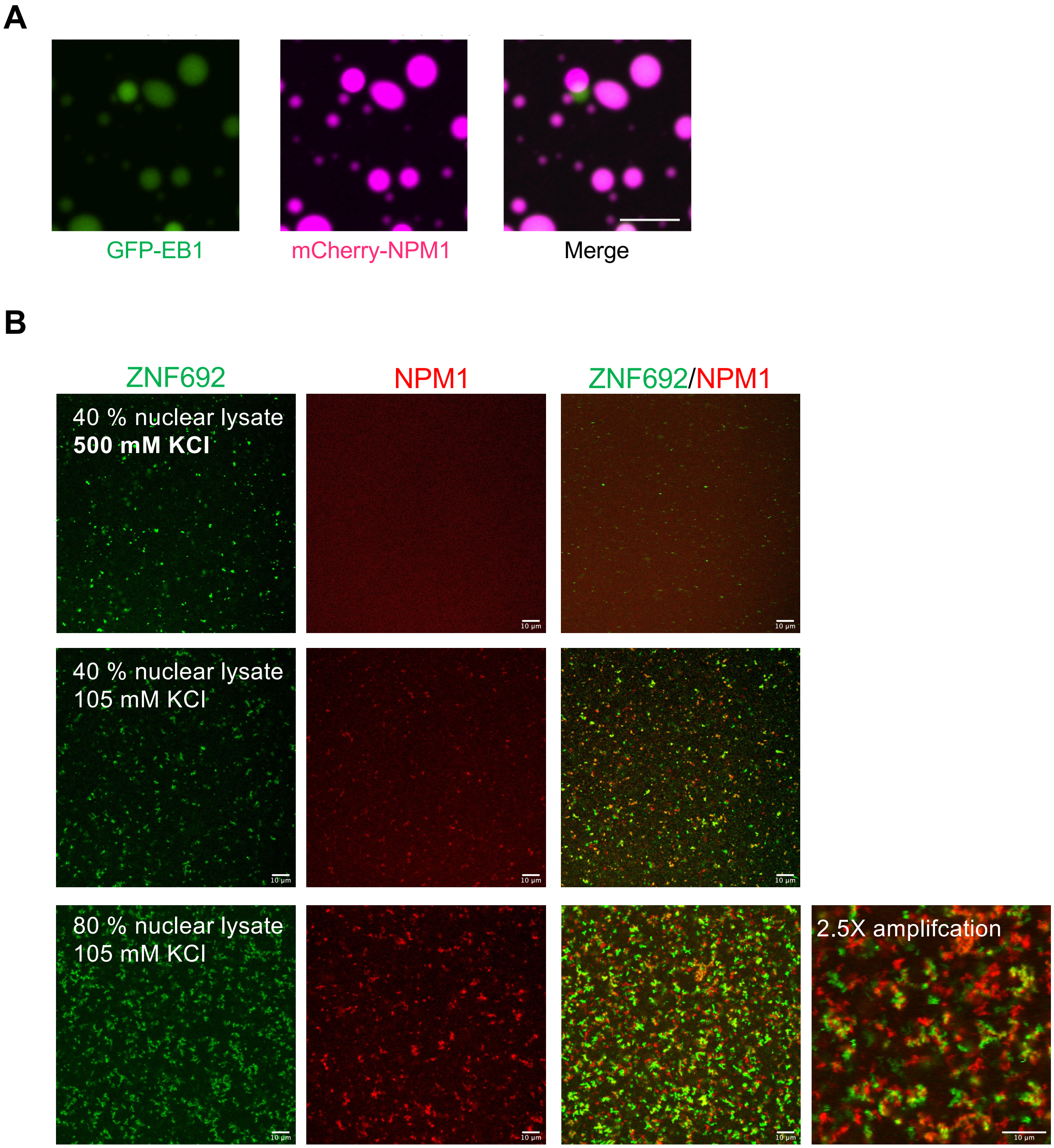
A
GFP-EB1
mCherry-NPM1
Merge
B
ZNF692
NPM1
ZNF692/NPM1
40 % nuclear lysate
500 mM KCl
40 % nuclear lysate
105 mM KCl
80 % nuclear lysate
105 mM KCl
2.5X amplifcation

Figure S2

A

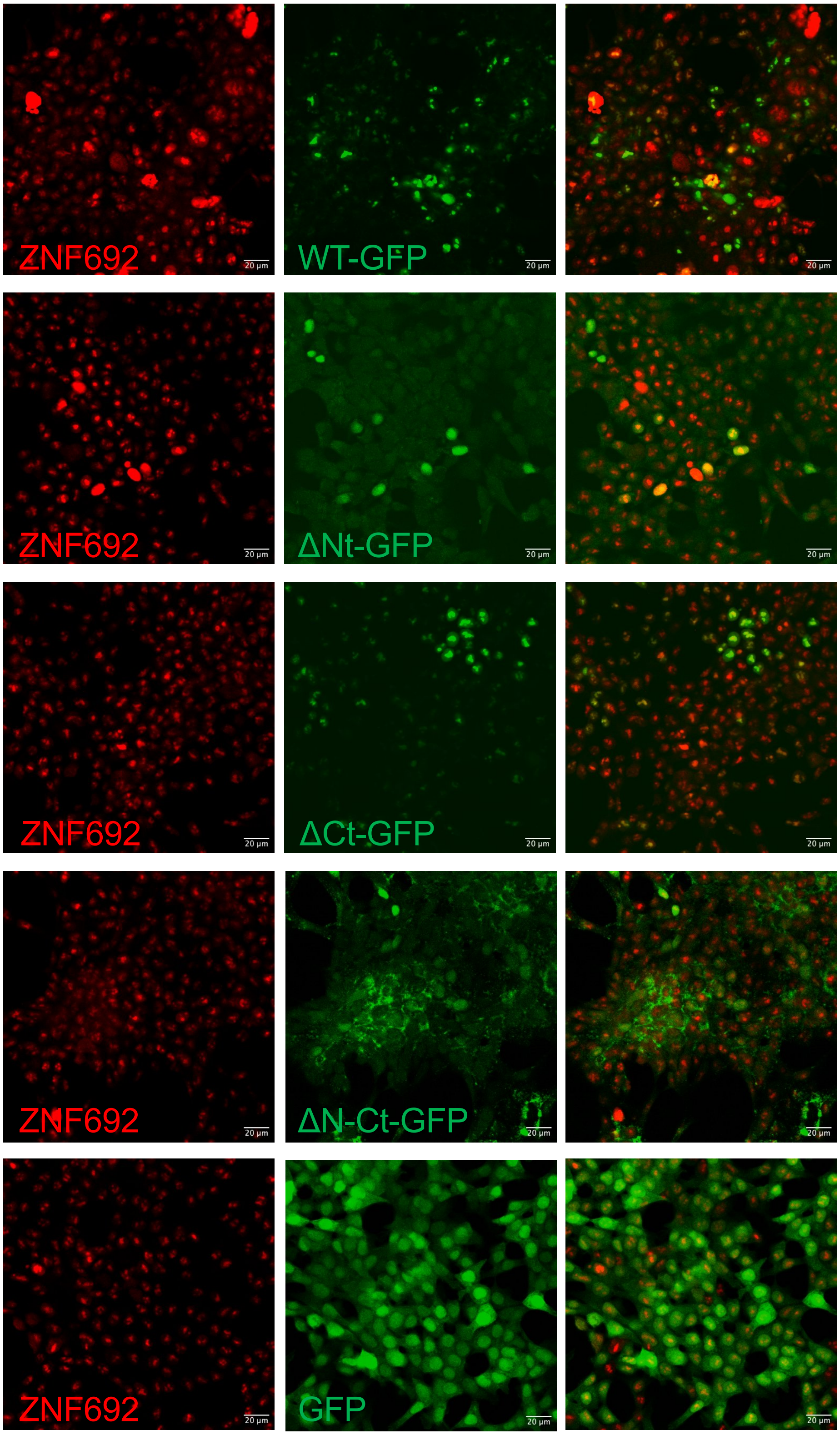
ZNF692
WT-GFP
ΔNt-GFP
ΔCt-GFP
ΔN-Ct-GFP
GFP
20 μm

B

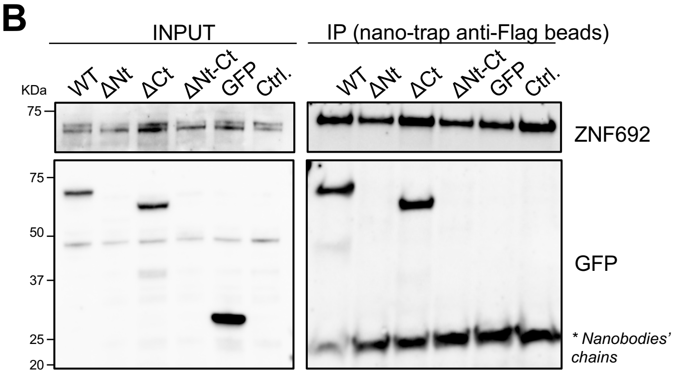
INPUT
IP (nano-trap anti-Flag beads)
WT
ΔNt
ΔCt
ΔNt-Ct
GFP
Ctrl.
KDa
75
50
37
25
20
ZNF692
GFP
* Nanobodies' chains

Figure S3

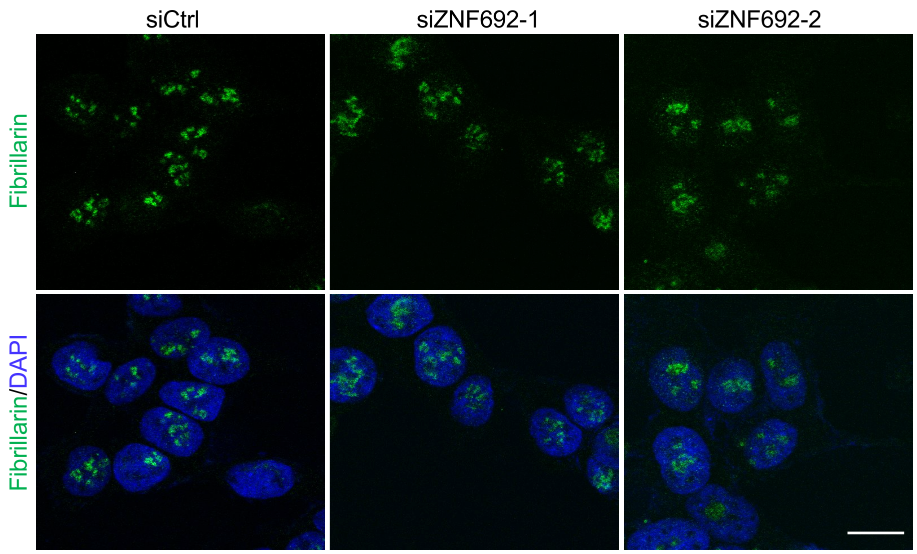

siCtrl
siZNF692-1
siZNF692-2
Fibrillarin
Fibrillarin/DAPI

Figure S4

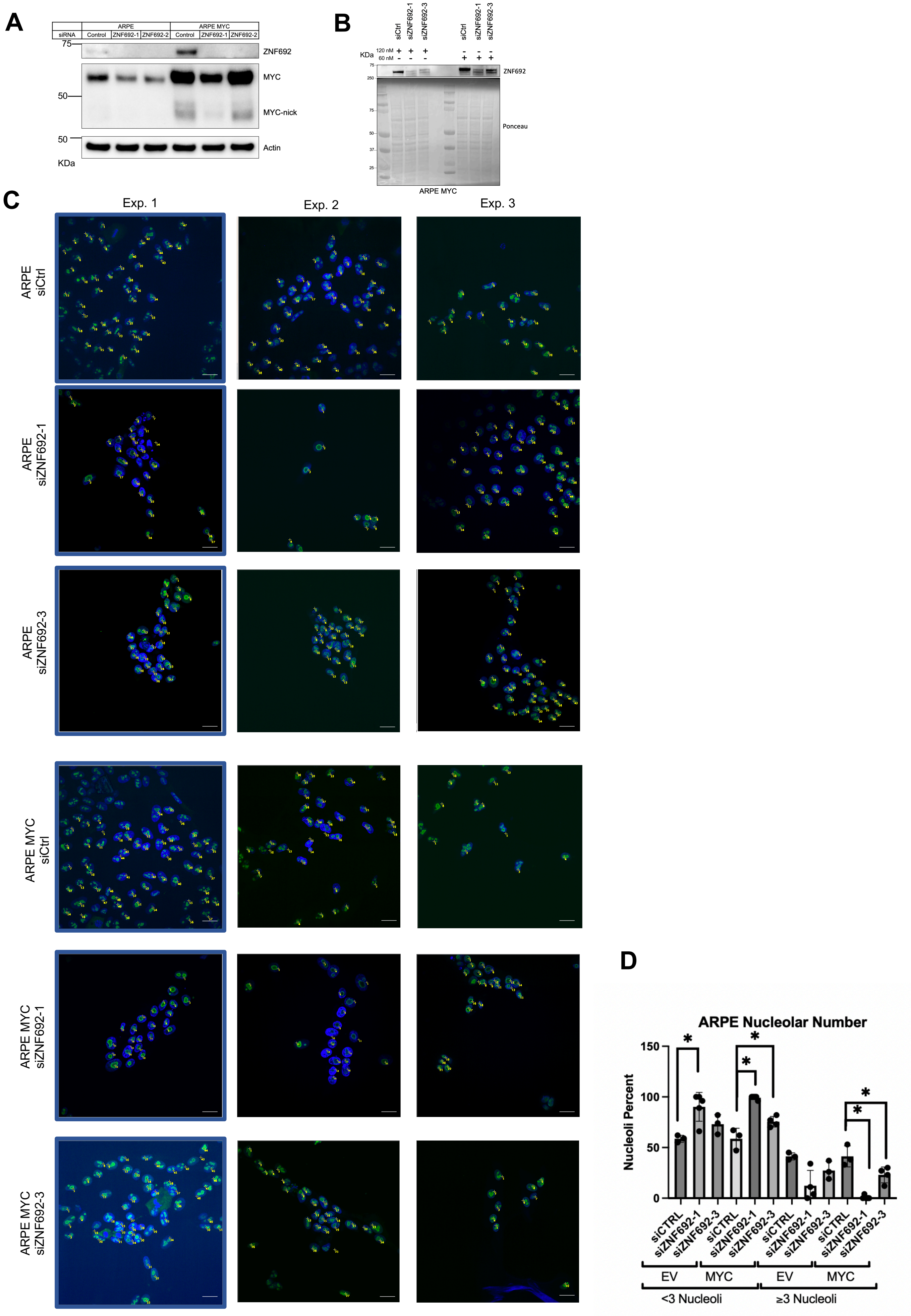
A
siRNA
ARPE
ARPE MYC
Control
ZNF692-1
ZNF692-2
75
50
50
KDa
ZNF692
MYC
MYC-nick
Actin
B
KDa
120 nM
60 nM
siCtrl
siZNF692-1
siZNF692-3
ZNF692
Ponceau
ARPE MYC
C
Exp. 1
Exp. 2
Exp. 3
ARPE siCtrl
ARPE siZNF692-1
ARPE siZNF692-3
ARPE MYC siCtrl
ARPE MYC siZNF692-1
ARPE MYC siZNF692-3
D
ARPE Nucleolar Number
Nucleoli Percent
150
100
50
0
siCTRL
siZNF692-1
siZNF692-3
EV
MYC
<3 Nucleoli
≥3 Nucleoli

Figure S5

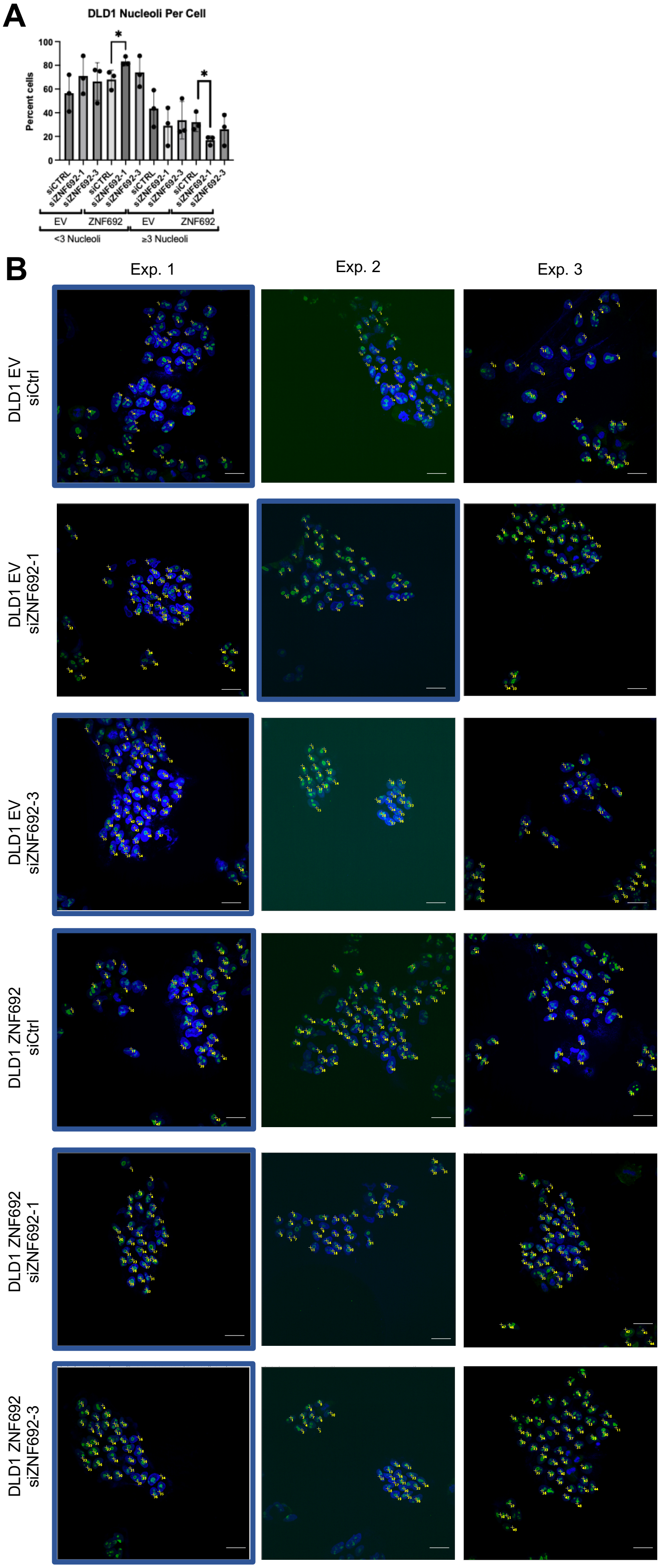
A
DLD1 Nucleoli Per Cell
Percent cells
100
80
60
40
20
0
*
*
siCTRL
siZNF692-1
siZNF692-3
siCTRL
siZNF692-1
siZNF692-3
siCTRL
siZNF692-1
siZNF692-3
siCTRL
siZNF692-1
siZNF692-3
EV
ZNF692
EV
ZNF692
<3 Nucleoli
≥3 Nucleoli
B
Exp. 1
Exp. 2
Exp. 3
DLD1 EV siCtrl
DLD1 EV siZNF692-1
DLD1 EV siZNF692-3
DLD1 ZNF692 siCtrl
DLD1 ZNF692 siZNF692-1
DLD1 ZNF692 siZNF692-3

Figure S6

ARPE EV

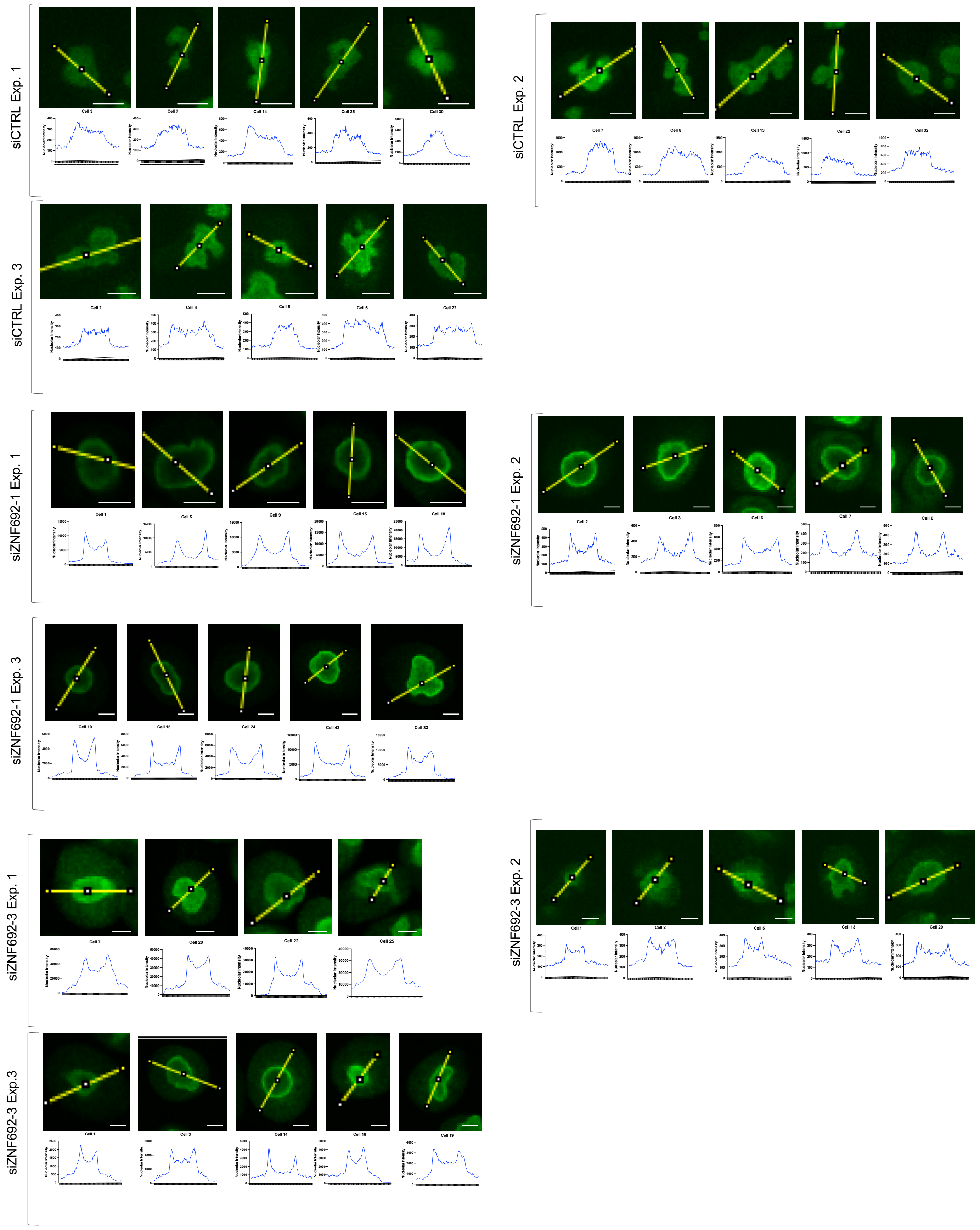

Figure S7

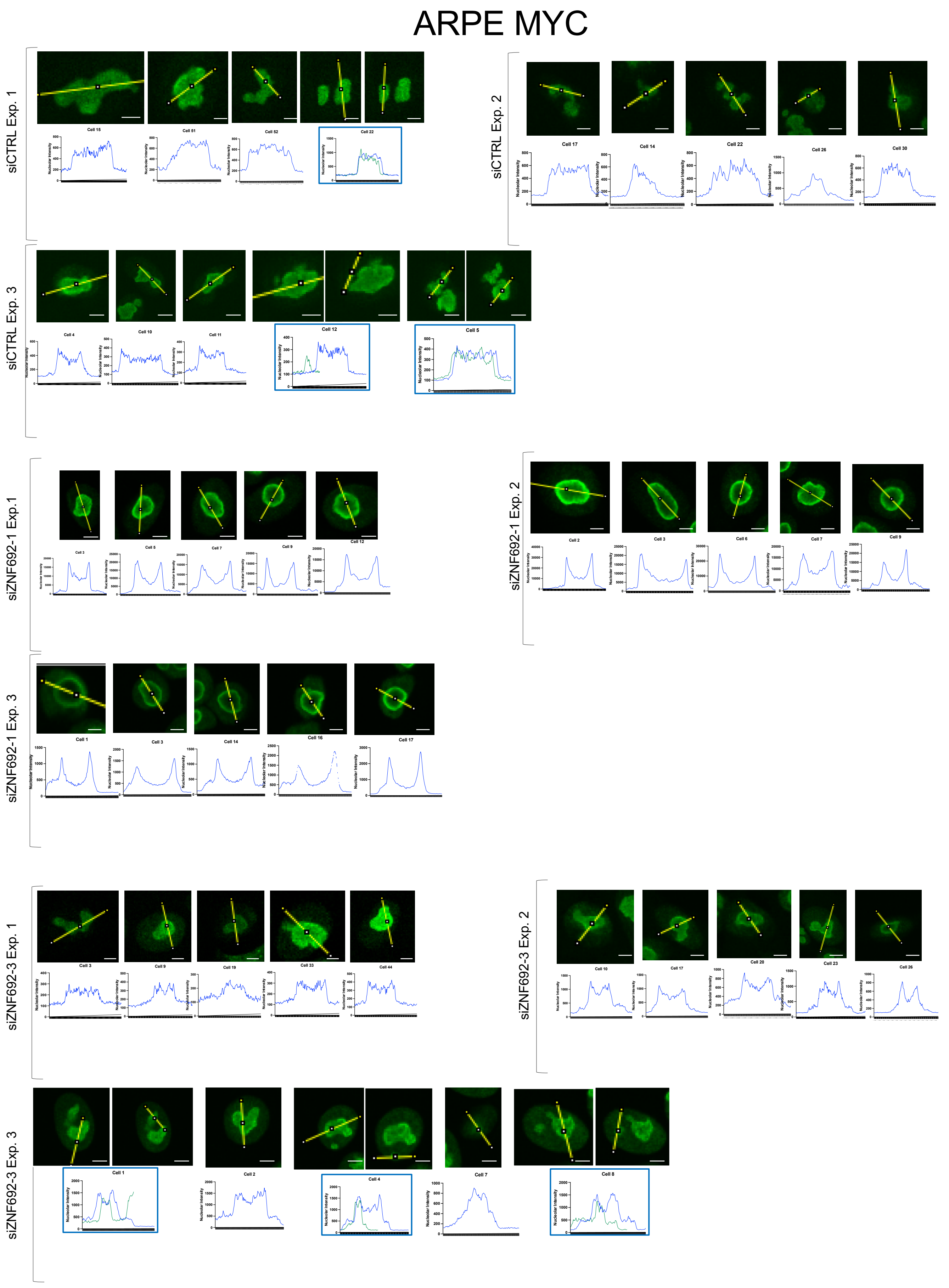
ARPE MYC
siCTRL Exp. 1
Cell 15
Cell 51
Cell 52
Cell 22
siCTRL Exp. 2
Cell 17
Cell 14
Cell 22
Cell 26
Cell 30
siCTRL Exp. 3
Cell 4
Cell 10
Cell 11
Cell 12
Cell 5
siZNF692-1 Exp.1
Cell 3
Cell 5
Cell 7
Cell 9
Cell 12
siZNF692-1 Exp. 2
Cell 2
Cell 3
Cell 6
Cell 7
Cell 9
siZNF692-1 Exp. 3
Cell 1
Cell 3
Cell 14
Cell 16
Cell 17
siZNF692-3 Exp. 1
Cell 3
Cell 9
Cell 19
Cell 33
Cell 44
siZNF692-3 Exp. 2
Cell 10
Cell 17
Cell 20
Cell 23
Cell 26
siZNF692-3 Exp. 3
Cell 1
Cell 2
Cell 4
Cell 7
Cell 8
Nucleolar Intensity

Figure S8

DLD1 EV

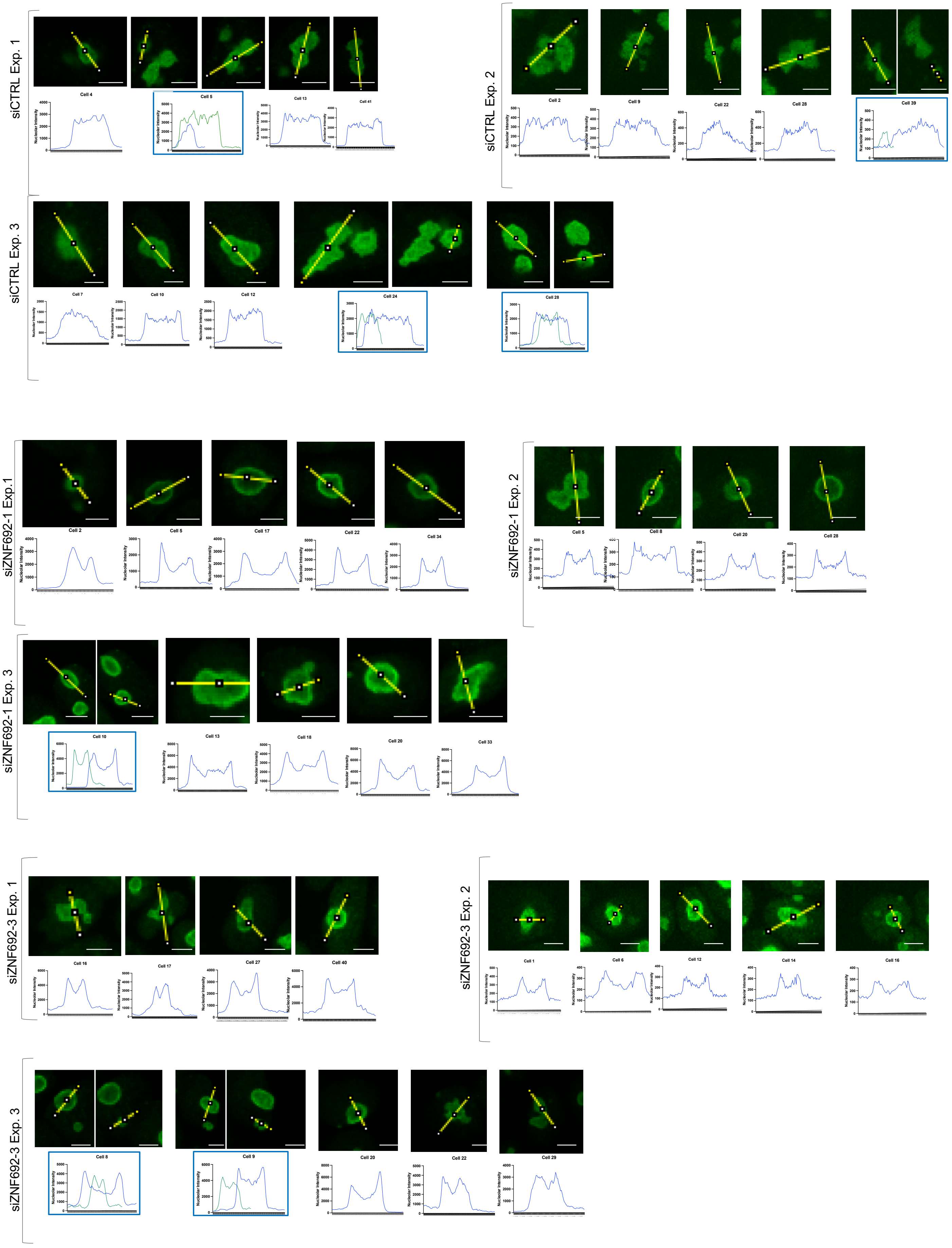

Figure S9

DLD1 ZNF692

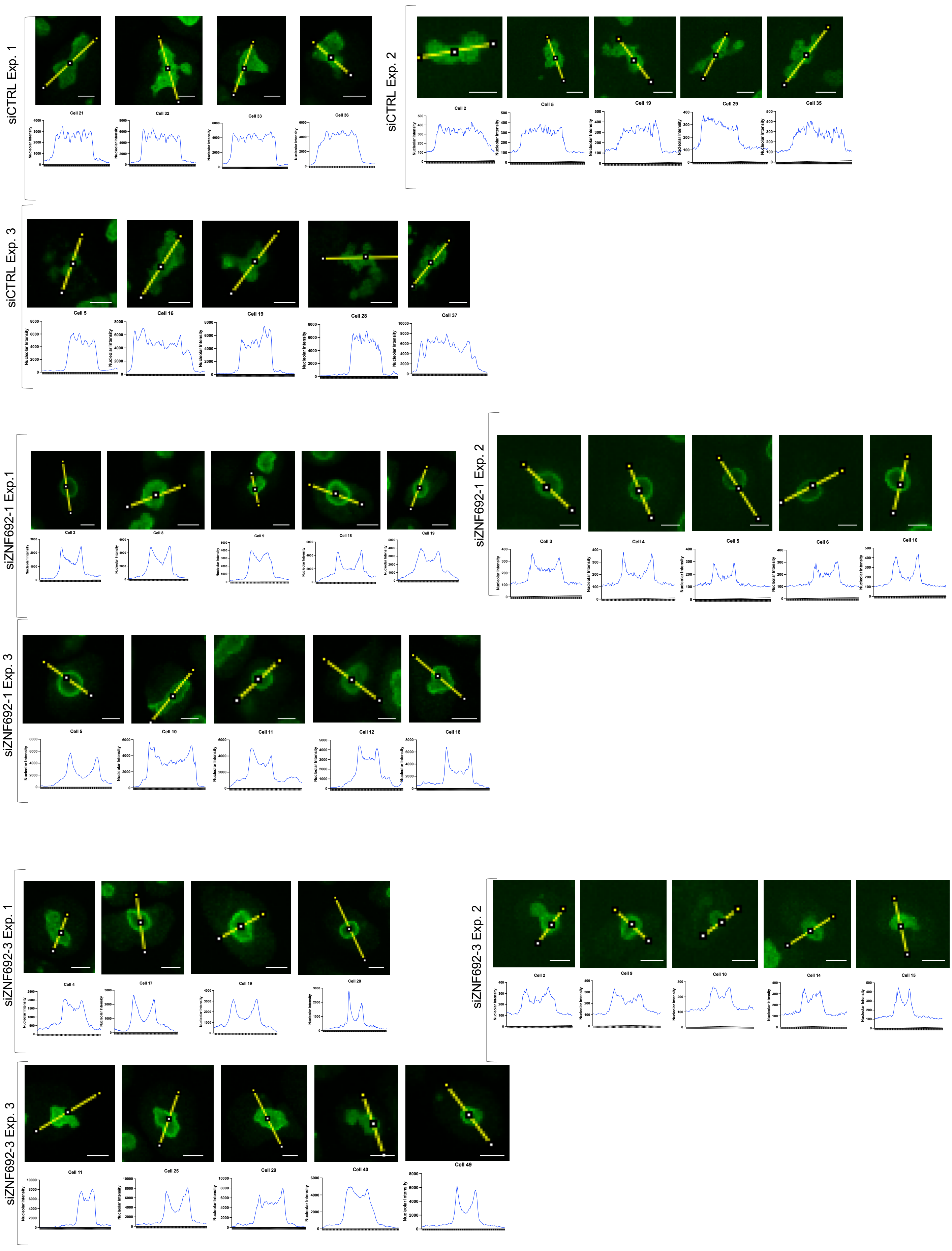

Figure S10
